# Efficacy prediction of noninvasive ventilation failure based on the stacking ensemble algorithm and autoencoder

**DOI:** 10.1186/s12911-022-01767-z

**Published:** 2022-01-31

**Authors:** Na Liang, Chengliang Wang, Jun Duan, Xin Xie, Yu Wang

**Affiliations:** 1 College of Computer Science, Chongqing University, Chongqing, 400000 People’s Republic of China; 2 Department of Respiratory and Critical Care Medicine, The First Affiliated Hospital of Chongqing Medical University, Chongqing, 400016 People’s Republic of China; 3 Chongqing Health Statistics Information Center, Chongqing, 401120 People’s Republic of China

**Keywords:** Noninvasive ventilation, SMSN model, Autoencoder, Efficacy prediction

## Abstract

**Background:**

Early prediction of noninvasive ventilation failure is of great significance for critically ill ICU patients to escalate or change treatment. Because clinically collected data are highly time-series correlated and have imbalanced classes, it is difficult to accurately predict the efficacy of noninvasive ventilation for severe patients. This paper aims to precisely predict the failure probability of noninvasive ventilation before or in the early stage (1–2 h) of using it on patients and to explain the correlation of the predicted results.

**Methods:**

In this paper, we proposed a SMSN model (stacking and modified SMOTE algorithm of prediction of noninvasive ventilation failure). In the feature generation stage, we used an autoencoder algorithm based on long short-term memory (LSTM) to automatically extract time series features. In the modelling stage, we adopted a modified SMOTE algorithm to address imbalanced classes, and three classifiers (logistic regression, random forests, and Catboost) were combined with the stacking ensemble algorithm to achieve high prediction accuracy.

**Results:**

Data from 2495 patients were used to train the SMSN model. Among them, 80% of 2495 patients (1996 patients) were randomly selected as the training set, and 20% of these patients (499 patients) were chosen as the testing set. The F1 of the proposed SMSN model was 79.4%, and the accuracy was 88.2%. Compared with the traditional logistic regression algorithm, the F1 and accuracy were improved by 4.7% and 1.3%, respectively.

**Conclusions:**

Through SHAP analysis, oxygenation index, pH and H1FIO_2_ collected after 1 h of noninvasive ventilation were the most relevant features affecting the prediction.

## Introduction

The total mortality of patients with noninvasive ventilation (NIV) failure is 23–27% [1, 2]. However, the mortality of patients with advanced NIV failure increases dramatically, ranging from 50 to 80%. Early prediction of noninvasive ventilation failure is of great significance for critically ill ICU patients to escalate or change the treatment. In this paper, we proposed how to build a good prediction model and analysed which features would influence the final prediction of using noninvasive ventilation on patients before or in the early stage (1–2 h). First, in the course of treatment of ICU patients, there were far fewer noninvasive ventilation failed samples collected in hospitals than successful samples. Due to the small number of failed samples, the prediction or recall of the classifier is reduced, which leads to easily missed diagnoses in clinical practice. Second, the prediction of a single classifier has a high deviation. In the face of complex diseases, hospitals need to solicit the opinions of doctors from different disciplines for comprehensive judgement to improve the diagnostic accuracy of patients and the adaptability of treatment options. Finally, most machine learning algorithms do not actively obtain time-series relationships of data, such as 1 h before and after treatment. However, these data are important for doctors to observe changes in diseases and judge the efficacy. In conclusion, it is very meaningful to build a good prediction classifier with interpretability and analyse the overall and individual features that affect predictions. In this paper, our contributions are as follows.

First, we proposed the SMSN model for the first time, including three stages: data processing, feature generation and modelling. Through experimental verification, the SMSN model was the best in AUC, F1, and accuracy performance measures, which improved the accuracy of prediction.

Second, we innovatively adopted an autoencoder model based on LSTM. It can actively extract time series data and automatically extract time series features by using LSTM’s ability to process time series and the autoencoder’s ability to extract features and thus adds them to the dataset to improve the accuracy of prediction.

Then, in view of the minority classes that are difficult to classify, we proposed an oversampling algorithm (modified SMOTE) for the first time. This kind of algorithm focuses more on classes with less data, promotes class balance and improves the sensitivity or recall of the classifier.

Finally, we adopt the stacking ensemble algorithm including three classifiers, including simple logistic regression, random forests and Catboost in nonlinear tree classifiers.

Noninvasive ventilation failure was defined as the requirement of intubation for invasive mechanical ventilation. However, for doctors with low seniority, they usually make incorrect judgements on when and what kind of treatment is appropriate due to lack of experience, resulting in the failure of treatment and operation. Therefore, machine learning algorithms assist doctors in making correct judgements, which is of great significance to improve the cure rate of patients and reduce mortality.

## Related research

### Traditional statistical methods

A study showed that data from June 2011 to June 2018 were collected from the First Affiliated Hospital of Chongqing Medical University (Chongqing, China), the First Affiliated Hospital of Xi’an Medical University (Xi’an, China) and People’s Hospital of Changshou Chongqing (Chongqing, China) in 2019. A total of 500 patients were randomly selected into the derivation cohort, and the remaining 323 patients were included in the internal validation cohort. Five influencing factor variables were chosen: heart rate, acidosis (assessed by pH), consciousness (assessed by Glasgow coma score), oxygenation and respiratory rate [1]. In conclusion, in high-risk patients identified by the HACOR score assessed at 1–2 h of NIV, early intubation is associated with decreased hospital mortality. However, the study was restricted to one disease of COPD, and there were only five variable factors in the study. Additionally, significant variable factors such as nursing were not considered. Chi-square and/or Fisher’s exact tests were used in the study, and the quantity was very limited. External verification of the study only came from and was simply performed by two hospitals with a lack of universality. Accordingly, the dependability of the conclusion requires further improvement.

### Machine learning

In a cooperative experiment conducted by the Chinese People’s Liberation Army General Hospital (Beijing, China) and Cornell University (Ithaca, New York, United States), the data of 43,336 patients gathered from three intensive care units in the United States were applied for model development. In addition, the data of 24,819 patients were from the test set, and 40 clinical variables were collected from each patient per hour, including 8 vital sign variables, 26 laboratory variables and 6 demographic variables. A total of 312 features were constructed per hour as input to the time-phased machine learning model for sepsis prediction [3]. LightGBM, an effective and efficient gradient boosting decision tree algorithm, was used to predict the risk of sepsis, and the proposed time-phased machine learning classifier for sepsis prediction was accurate in the experiment. However, it did not sufficiently mine the time series data collected at different times.

### Ensemble learning

Ensemble learning is a combined decision-making based on basic machine learning algorithms, which completes the learning task [4, 5] by training multiple base models and combining them. Generally, ensemble learning algorithms, including the bagging algorithm [6], boosting algorithm [7] and stacking ensemble algorithm [8], have better performances than single machine learning algorithms. The bagging algorithm obtains the training set of each base model by bootstrap sampling methods, thereby having different base classifiers. The boosting algorithm trains iteratively by increasing the weight of misclassified instances of the previous weak classifier and obtains a strong learner. The stacking ensemble algorithm takes the prediction of the base classifier as new features, trains the original classifier with these new features, and obtains the predictions.

Through experiments, Statnikov et al. found that random forests of the decision tree classifier based on an ensemble learning algorithm carried out the best performance in cancer classification [9]. In the EEG classification task, Hosseini et al. [9, 10] developed a random subspace ensemble method using a combination of various methods as the base classifier. Dongxiao et al. [11] proposed a method that adopted extreme gradient boosting (XG boost) for breast cancer recurrence prediction. Pooja Tukaram Dalvi et al. discovered that the stacking ensemble algorithm had the highest performance in anaemia classification and detection.

### Data imbalance

In the medical datasets [9, 12], the data were mainly composed of normal samples, and a few of them were abnormal samples, leading to medical data imbalance. For data imbalance, oversampling algorithms are repeatedly used to generate new minority instances to achieve class balance. The SMOTE algorithm [11–13] is a classic oversampling algorithm, and the generation of its new instances is interpolated in accordance with the minority sample point x and the k nearest instance points to x. Compared with the random duplication of minority instances, the SMOTE algorithm balances the dataset and dramatically reduces the randomness in the sampling process. Rahman et al. [11, 14] compared common oversampling algorithms and found that the SMOTE algorithm [11–13] achieved excellent performances in multiple medical datasets. On the basis of the SMOTE algorithm, the Borderline-SMOTE algorithm divides the minority instances into three categories in line with most of the instances from the adjacent points, which are noise class, boundary class, and security class. KMeans SMOTE [12, 13, 15] combines k-means and the SMOTE algorithm to effectively overcome the problem of imbalance between and within classes, as well as the dependency of the nearest neighbours.

### Interpretability of medical classification

At present, in the face of increasing noninvasive respiratory data, machine learning can make use of its big data capability to make highly accurate predictions. However, some complex machine learning algorithms have a major disadvantage in the medical field: their similarity to a black box conceals the reasons for decision-making. This challenge makes the medical field extremely cautious of the application of machine learning. Accordingly, it requires not only high accuracy of the model but also strong interpretability to obtain numerous applications of deep learning in the medical field more quickly. For this purpose, many researchers have studied the interpretability of machine learning. LIME [16], SHAP [12, 13, 15, 17, 18] and other tools have been constantly proposed in research on the interpretability of traditional machine learning.

## Prediction models

### Based on stacking ensemble algorithm

#### Data sources

The data of this study were derived from the electronic medical record database of the First Affiliated Hospital of Chongqing Medical University (Chongqing, China) from January 1, 2011 to January 25, 2019. As shown in Table 1, the data of 2495 severe patients were collected, and 13 disease diagnoses were involved. There were 1122 cases of AECOPD (45%), 652 cases of pneumonia (26.1%), 197 cases of ARDS (7.9%), and 524 cases of other diagnoses, accounting for 21%. The data information had four latitudes, which were the basic information parameters of patients, covering names, genders, ages and diagnoses; indices related to noninvasive ventilation failure, including H0 oxygenation index, H1 oxygenation index, H0GCS, H1GCS and other data recorded every hour; outcomes data: whether noninvasive ventilation failed, and APACHE score; past medical history data: whether patients had hypertension, diabetes, chronic liver and kidney diseases and cardiac insufficiency, etc. These data were recorded at hourly intervals, and the values of the above patient data were recorded every 1 h after hospitalization. In addition, the data were divided into quartiles by box plots. The data with the default distance exceeding the average value of 3δ were abnormal data. The abnormal values or the data that were not collected were regarded as missing values, which were replaced by medians.

**Table1:** Characteristics of the enrolled patients

Item	Value or number
Age, years	69 ± 14
Male	N = 1794
APACHE II	16 ± 5
NIV failure	N = 740
Diagnosis	
AECOPD	N = 1122
Pneumonia	N = 652
ARDS	N = 197
Sleep apnoea-hypopnea syndrome	N = 65
Asthma	N = 70
Heart failure	N = 50
Bronchiectasis	N = 46
Sepsis	N = 44
Pulmonary embolism	N = 39
Pulmonary tuberculosis associated sequelae	N = 35
Chest wall deformity	N = 22
Obesity-hypoventilation syndrome	N = 6
Chronic thoracic sequelae	N = 2
Others	N = 145
Underlying disease	
Hypertension	N = 889
Diabetes mellitus	N = 493
Chronic liver disease	N = 53
Chronic kidney disease	N = 104
Chronic heart disease	N = 201
Chronic lung disease	N = 1459
Immunosuppression	N = 100
Solid tumour	N = 238
Variables collected before NIV	
GCS	14.6 ± 1.2
Systolic blood pressure, mmHg	137 ± 26
Diastolic blood pressure, mmHg	81 ± 16
Heart rate, beats/min	134 ± 23
Respiratory rate, breaths/min	31 ± 7
pH	7.36 ± 0.11
FiO_2_	0.42 ± 0.15
PaCO_2_, mmHg	57 ± 26
PaO_2_, mmHg	72 ± 39
PaO_2_/FiO_2_	178 ± 87
Variables collected after 1 h of NIV	
Tidal volume, mL	413 ± 160
Minute ventilation, L	11.2 ± 6.6
Inspiratory pressure, cmH_2_O	15 ± 4
Expiratory positive airway pressure, cmH_2_O	6 ± 2
GCS	14.6 ± 1.2
Systolic blood pressure, mmHg	127 ± 23
Diastolic blood pressure, mmHg	74 ± 14
Heart rate, beats/min	104 ± 23
Respiratory rate, breaths/min	27 ± 7
pH	7.39 ± 0.09
FiO_2_	0.50 ± 0.16
PaCO_2_, mmHg	54 ± 22
PaO_2_, mmHg	95 ± 40
PaO_2_/FiO_2_	205 ± 89

Among them, 80% of 2495 patients (1996 patients) were randomly selected as the training set, and 20% of these patients (499 patients) were chosen as the testing set. The Ethics Committee approved the research protocol. Informed consent was obtained from patients or their family members.With several teaching hospitals and an inherent advantage in patient data collection, the hospital also has an advantage in machine learning.

## Research objects

According to the inclusion and exclusion criteria, 2495 patients were selected from the database as the research subjects, with a total of more than 99,000 data points. The inclusion criteria were patients who were admitted to the intensive care unit, patients who completed the required inspection indicators after hospitalization, and patients whose data were collected at hourly intervals. The exclusion criteria were noncritical patients who were not admitted to the hospital, patients with a large number of missing examination results after hospitalization, and patients whose data were not collected at one-hour intervals.

### Proposed model framework

**Fig. 1:**
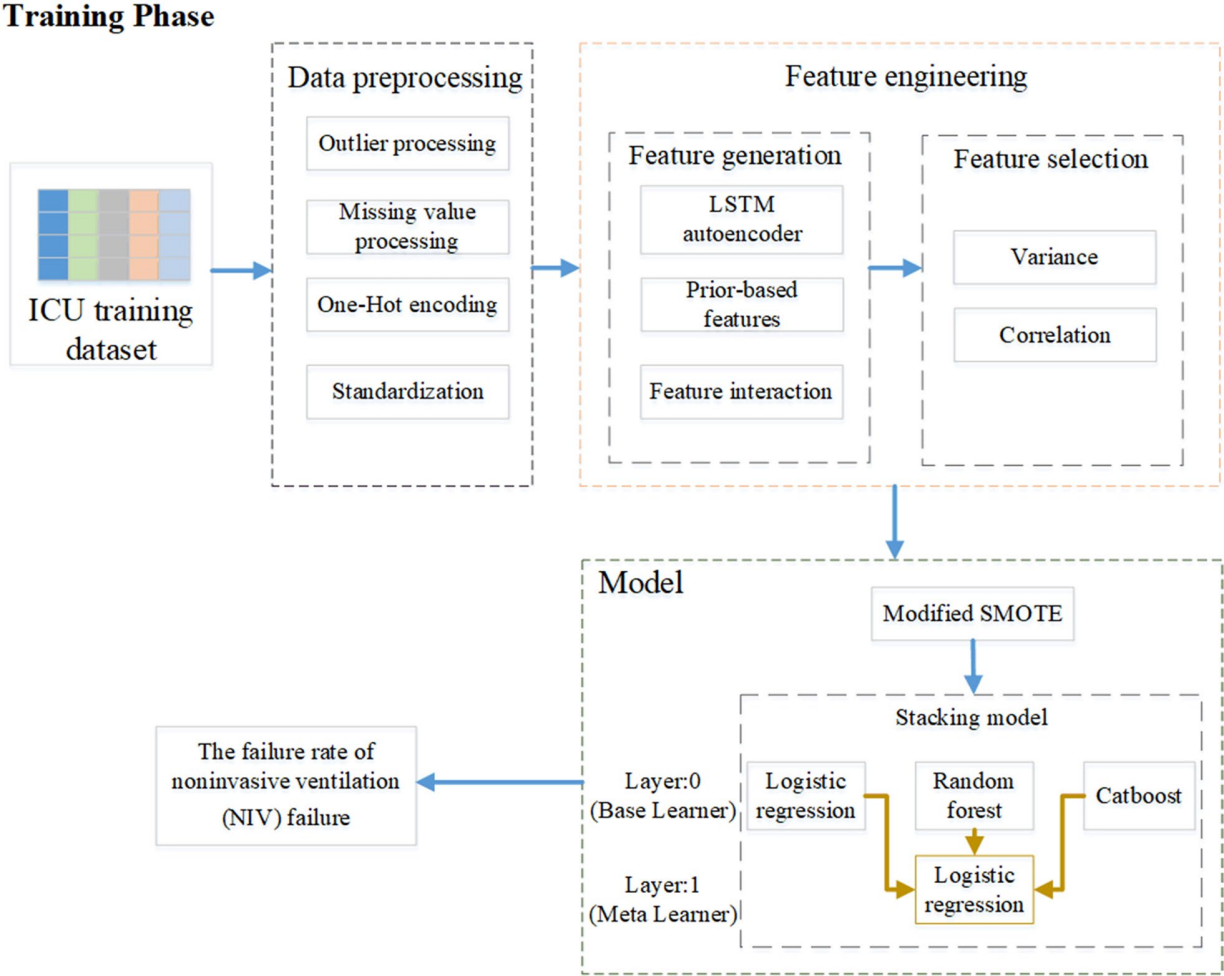
The training flowchart of SMSN model

The proposed SMSN model, as shown in Fig. 1, includes the modified SMOTE algorithm to deal with data imbalance and the LSTM autoencoder to exact time series features. The stacking ensemble algorithm carries out ensemble learning and combines multiple machine learning classifiers to improve the prediction accuracy. First, data preprocessing was carried out in the dataset, including missing value processing, outlier processing, and data standardization. Second, feature engineering was executed in the dataset, covering feature generation and feature selection. To make full use of the features of the time series in the data, the LSTM autoencoder was used as the extraction model of the features of the time series. Ultimately, a modified SMOTE model was established to balance the minority classes and the majority classes. At the same time, a stacking ensemble algorithm was made to improve the prediction accuracy by combining logistic regression, random forest and the Catboost algorithm. The three components will be introduced in sequence in the following chapters.

### Data preprocessing

There were outliers in the collected data. In this paper, box plots were applied to divide the data into quartiles. When the data with the default distance exceeded the average value of 3δ, they were deemed abnormal data. These abnormal data or uncollected data were treated as missing values. To increase the stability of the filling of missing values, this paper did not make use of the average value but the medians to fill the missing values.

Distance-based machine learning algorithms, such as KNN [19], logistic regression [20, 21] and SVM [9, 12, 13, 15, 17], are sensitive to the scale of each dimension of the input data, so preprocessing is required. In this paper, the z score standardization algorithm was selected to preprocess the data. Assuming that the input data, its mean value, and the standard deviation are x, $\overline{x}$ and *σ*, respectively, the transformation is as follows:

1 $$x^{\prime } = \frac{{x - \overline{x}}}{\sigma }$$

**Fig. 2:**
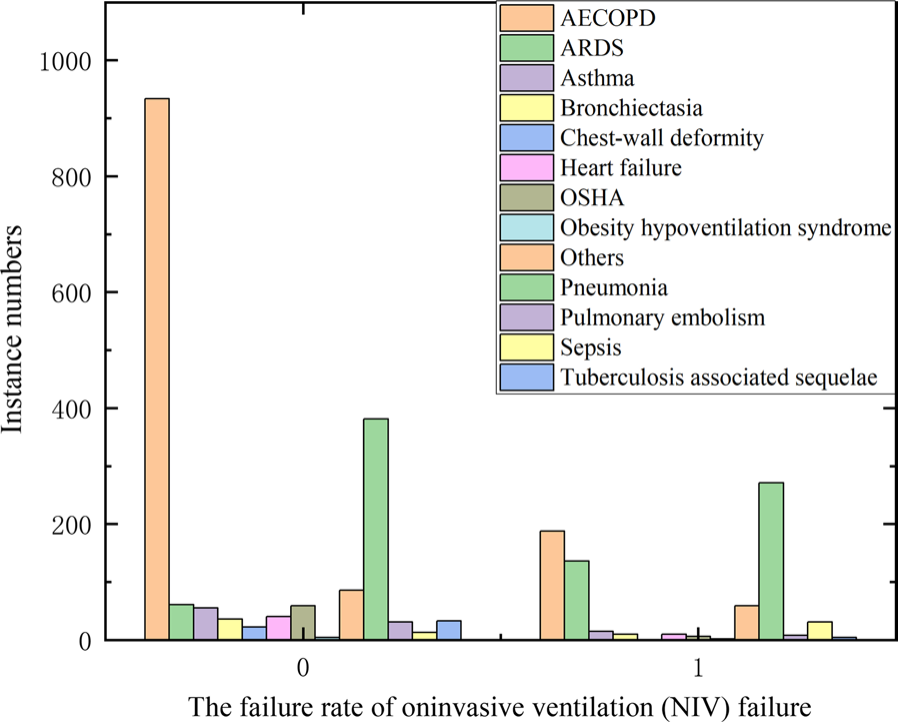
Causes and categories of respiratory failure

In the dataset collected in this paper, the causes of respiratory failure were characterized by categorical variables, which were not evenly distributed among various categories and had a high correlation with predicting the treatment failure of the variables. As shown in Fig. 2, when the reason for respiratory failure is AECOPO, the probability of treatment failure is low. When respiratory failure is caused by ARDS, the rate of treatment failure is high. In this paper, OntHot encoding was chosen as the categorical variables encoding method. In addition, the number of some diseases in the sample is less than 20. In the process of onthot encoding, these diseases are classified into other diseases.

### Feature engineer

#### Feature generation

Feature generation uses the existing features to generate new features and fully mine the information of the dataset to improve the prediction ability of the model. The new features need to be validated, and domain knowledge, industry experience and mathematical knowledge are required to comprehensively consider the validity of the features. In this paper, three methods were used for feature generation in accordance with the features of the time series correlation of data and clinical expert knowledge.

**Fig. 3:**
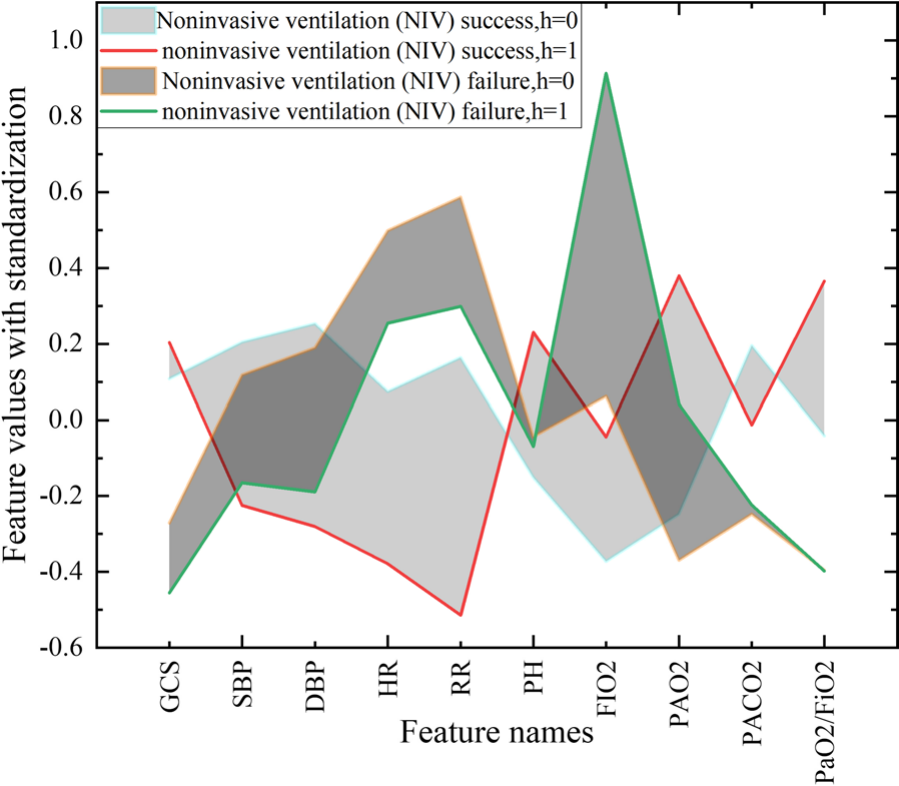
Visual time series

First, in the ICU dataset, respiratory detection data of each patient at two time points were collected to form a time series. The performances of traditional machine learning classifiers were reduced because they did not take advantage of the features of time series in the data. As shown in Fig. 3, the mean values of different detection features of the two categories at two time points h = 0 and h = 1 are visualized. Compared with patients with successful treatment, GCS, PaCO_2_ and oxygenation index features were at lower levels in patients with failed treatment, while HR, RR and FIO_2_ features were at higher levels in patients with failed treatment. In addition, PaO_2_ and oxygenation index features increased significantly when h = 1 in patients with successful treatment. Meanwhile, the HR, RR and FIO_2_ in patients with failed treatment increased remarkably at h = 1. The change in detection features was highly correlated with the prediction over time.

**Fig. 4:**
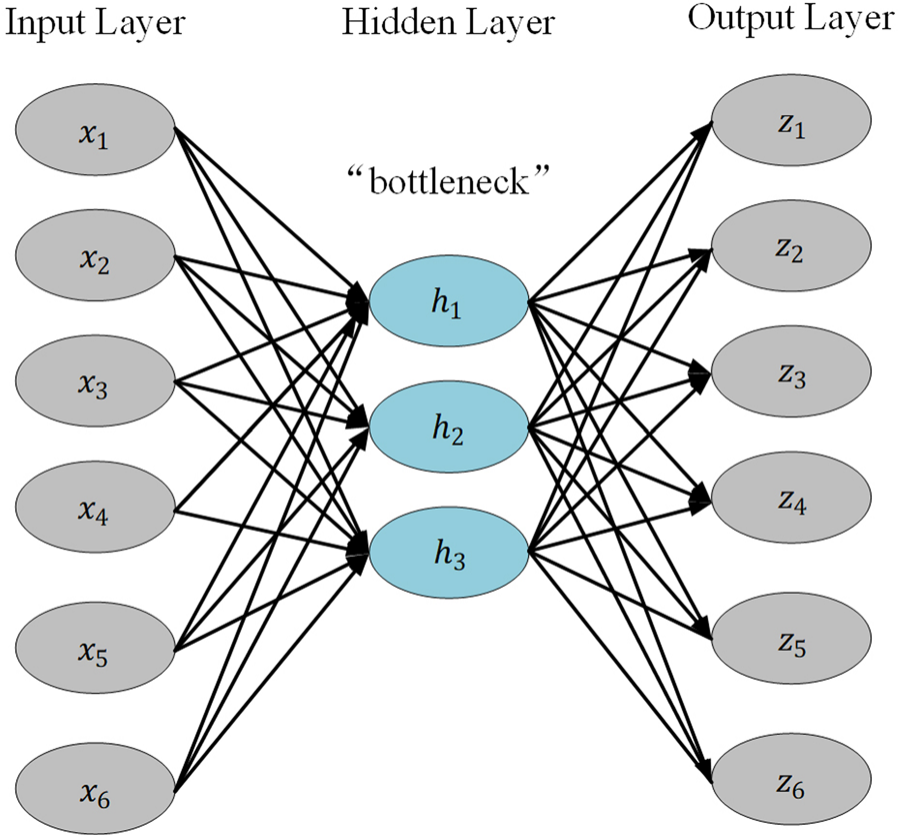
The architecture of the aotoencoder

**Fig. 5:**
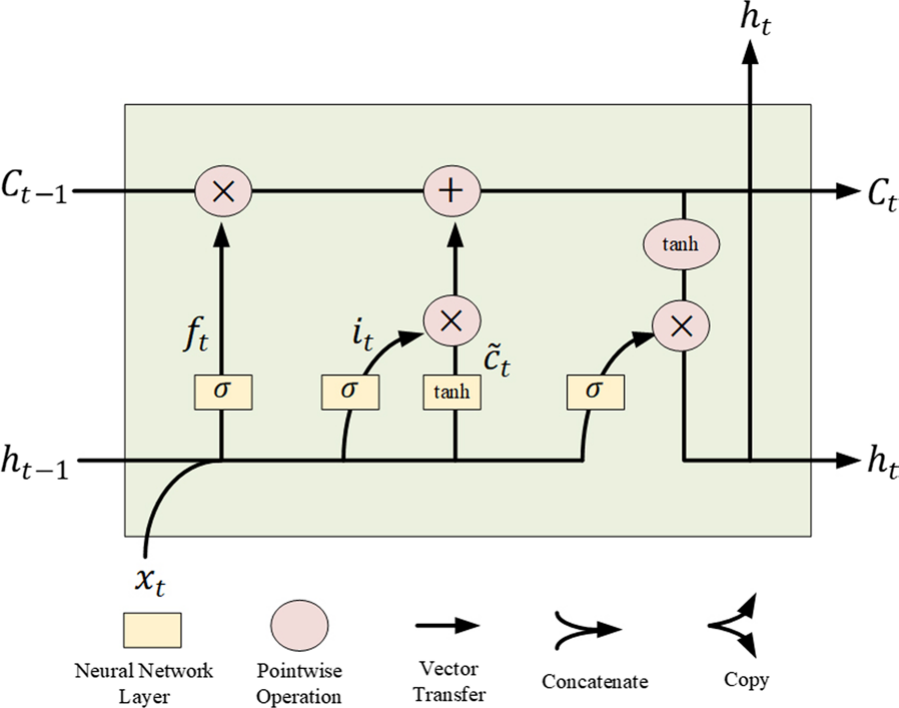
The structure of the LSTM network

In the feature engineering stage, an LSTM autoencoder model was used to extract the time-series features of the data sampled at two time points. As shown in Fig. 4, the variable **x** represents the raw data, which are treated as nodes in the input layer. It is encoded to the dimension-reduced vector h, which represents the nodes from the hidden layer. Then, the vector h shows that the dimensions of the input and output layers of the autoencoder are the same, and the encoder in the hidden layer automatically extracts the most useful features and restores the extracted features to raw data. The LSTM network could be framed as a chain of repeating modules where each module corresponds to a time point. A detailed structure is shown in Fig. 5. The key component of the chain is the cell state, which stores historical information. At each time point, the information is updated and transmitted under the interaction of 3 gates: forgetting gate, input gate, and output gate. Every one of them is a sigmoid neural network layer. LSTM is good at processing time-series data because of its memorizing historical information. In this paper, the autoencoder model based on LSTM was adopted in combination with LSTM processing time series data and autoencoder automatic feature extraction.

**Fig. 6:**
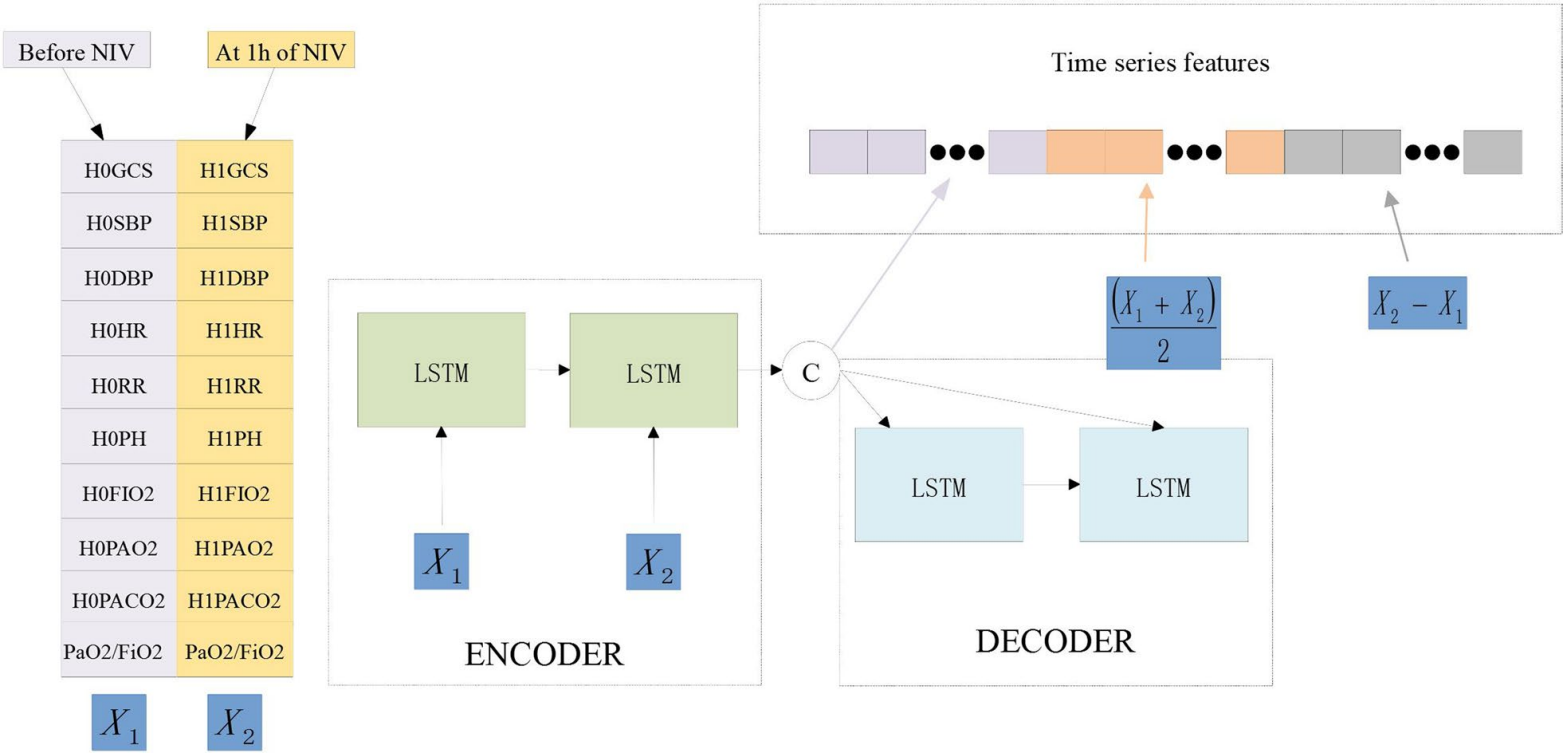
Extracting the features of time series

As shown in Fig. 6, we manually extracted the mean value and differential value of the features of two time points as new features and then used deep learning technology to automatically extract the features of the time series.

Attending physicians with rich clinical experience have formed expert formulas to generate prior features, as shown in Table 2. In addition, the feature crosses technique was used to multiply pairwise features to generate new features.

**Table 2:** Expert experience features

Before NIV (H0)	After 1 h of NIV (H1)	Unit
Oxygenation index/H0RR	H1oxygenation index/H1RR	mmHg/breath/min
Age*H0RR	Age*H1RR	year*breath/min
H0RR*H0HR	H1RR*H1HR	breath/min*beat/min
H0RR*H0HR*H0FIO_2_	H1RR*H1HR*H1FIO_2_	breath/min*beat/min

*NIV* noninvasive ventilation, *RR* respiratory rate, *HR* heart rate, *FIO*_*2*_ fraction of inspired oxygen

H0 means the data collected before NIV. H1 means the data collected after 1 h of NIV

#### Feature selection

Making new features requires considering their effectiveness to prevent the generation of meaningless features. Feature selection is a technology that reduces the dimensions of input variables and the runtime of the machine learning classifier and improves the prediction accuracy. The feature selection algorithm with low variance filtering is a simple method that calculates the variance corresponding to each feature value in the sample. If it is lower than the threshold value, it will be filtered (rejected). In addition, the Pearson correlation coefficient between features and tags is used for feature selection, which can filter irrelevant features. In this paper, variance and Pearson correlation coefficients were used to filter some features and retain the features with the highest correlation with the model.

## Model

### Modified SMOTE

The SMOTE algorithm is a classic oversampling algorithm. The generation of new instances is determined by the interpolation between the minority class instances and the k near them. Therefore, the SMOTE algorithm relies on neighbour k in the process of generating new instances and can only obtain the optimal value of k through repeated tests in the dataset. In addition, the use of the SMOTE algorithm will affect the data distribution of minority instances. If there is an isolated point in the minority classes in the dataset, there are no minority instance points nearby. Supposing that the particularity is not considered, the SMOTE algorithm will also generate new instance points near the isolated points, which will change the original data distribution to a large extent. At the same time, the SMOTE algorithm treats all minority instances equally. In classification, some misclassified minority instances are called hard instances, and correctly classified samples are called easy instances. Additionally, the SMOTE algorithm does not pay more attention to hard instances.

Modified SMOTE was proposed in this paper, which was more focused on hard samples than Borderline-SMOTE and k-means SMOTE while maintaining the advantages of Borderline-SMOTE and k-means SMOTE. The modified SMOTE algorithm first used the SVM classification algorithm to obtain a set of minority error instances. Then, it removed all isolated instances from the set of minority error instances to obtain a new set of instance points. Subsequently, clustering was performed by using the k-means algorithm in the instance points set, and N clusters were obtained. Eventually, the centre of clustering and the instance in the cluster were used to generate new instance points. The modified SMOTE algorithm procedure is presented in Algorithm 1:
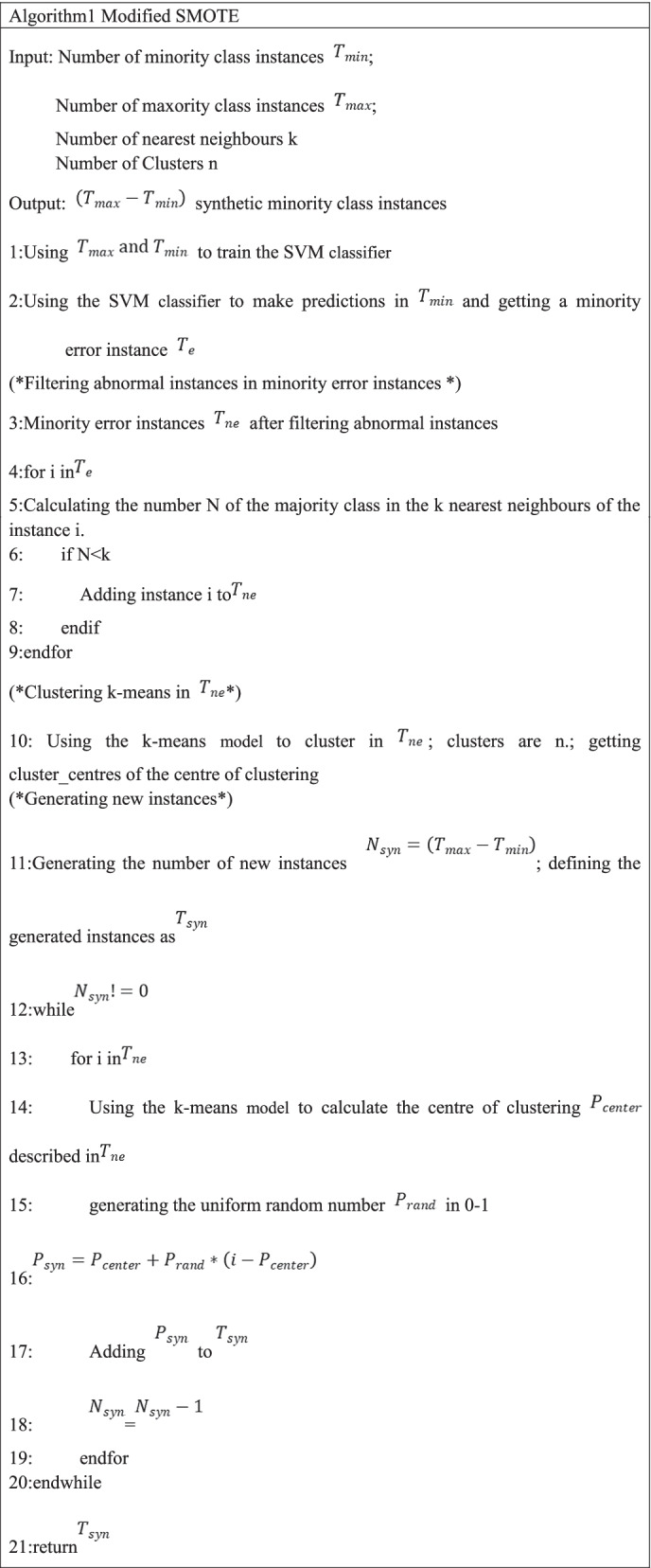


### Predictionsing model

In the model stage, a stacking integrated learning algorithm was adopted, which integrates logistic regression, random forest and Catboost. Logistic regression becomes a binary classification algorithm by adding a sigmoid function based on linear regression. The binary classification algorithm needs to make predictions in the range of [0, 1], while linear regression can only make predictions in the domain of real numbers. In this case, a sigmoid function is added to the linear regression to enable it to make predictions within the required range. Random forest establishes a forest in a random way. The forest is composed of many decision trees, and there is no correlation between them. Because of its randomness, it has strong anti-noise ability and is not easy to overfit. Catboost is a GBDT framework based on oblivious trees, and it has fewer parameters and high accuracy and supports category variables. Its greatest advantage is the efficient processing of category features. It was enhanced based on the XGBOOST algorithm, and it had a tremendous advantage in processing category features.

As shown in Fig. 1, the proposed prediction model used Catboost [22], random forests and logistic regression as base learners, and logistic regression as the meta learner. The prediction probabilities of each base learner were connected to form a new training set to train the meta learner. There were considerable differences among the selected classifiers, and stacking was able to make full use of the advantages of each learner to improve the performance of the classifier.

## Results

### Performance measures

The task of predicting the probability of noninvasive respiratory failure was evaluated. To improve the comprehensive and objective evaluation of the classifier, five different evaluation indices were used to evaluate the experiment. The basic terms of the confusion matrix will be introduced first. Positive class instances of true positives (TPs) are recognized as the positive classes; negative class instances of false-positives (FPs) are recognized as the positive classes; positive class instances of false negatives (FNs) are recognized as the negative classes; and negative class samples of true negatives (TNs) are recognized as the negative classes. The specific five Performance Measures are as follows:

Accuracy refers to the quantity of instances or the total number of instances that are predicted correctly. Accuracy represents the overall classification performance of the classifier, and its formula is as follows:

2 $${\text{Acc}} = \frac{TP + TN}{{TP + FP + TN = FN}}$$

Precision represents the ratio of the true positive classes in the instances that are judged to be the positive classes. It measures the number of errors of the positive classes predicted by the model are wrong. The formula is as follows:

3 $${\text{Precision}} = \frac{TP}{{TP + FP}}$$

Recall represents the ratio of instances that are judged to be the positive classes in the positive class instances, and it measures the model’s ability to recognize the positive classes.

4 $${\text{Recall}} = \frac{TP}{{TP + FN}}$$

The area under the receiver operating characteristic curve (AUC) was calculated. The receiver operating characteristic curve (ROC) represents the false-positive rate (FPR) and true positive rate (TPR).

The score of F1-Meature (F1) is a special case of F-Meature, which is a weighted sum of Precision and Recall and is suitable for the comprehensive judgement of the classifier. The F1 score expression is as follows:

5 $${\text{F}}1 = \frac{{2 \times {{Precision}} \times {{Recall}}}}{{{{Precision}} + {{Recall}}}}$$

As shown in Table 3, the logistic regression classifier was used in this paper to conduct experiments under the three conditions of the original features, the manual features, and all features. It was observed that when the original features were used, the classification accuracy was 0.869. When features other than the deep features were added, including features generated by the feature crosses technique, features of prior knowledge, and the feature mean of the two time points, the classification accuracy was increased by 0.05. When all features were used, the classification accuracy was 0.878; that is, when deep features were added, the classification accuracy of the model was increased by 0.04 compared to that without adding deep features. After the deep feature is added, the recall is significantly improved, which shows that the time series feature is very meaningful for the prediction of noninvasive respiratory failure.

**Table 3:** Classification experimental results of different features

Features	Accuracy	AUC	F1	Precision	Recall
The original features	0.869	0.905	0.747	0.872	0.653
The manual features	0.874	0.907	0.753	0.889	0.653
All features	0.878	0.907	0.77	0.864	0.694

By comparing and adding the generated features and the original features, it can be shown that the generated features can improve the performance of the classifier.

As shown in Table 4, common oversampling algorithms, including random oversampling, SMOTE, Borderline SMOTE, SVM SMOTE, and the proposed modified SMOTE, were used in this paper to carry out classification experiments based on the logistic regression model. According to Table 3, the SVM SMOTE model had the best accuracy and precision scores. The proposed modified SMOTE was optimal in AUC, F1 and recall indices, indicating that modified SMOTE could identify more minority classes.

**Table 4:** Classification experimental results of different oversampling algorithms

Classifiers	Accuracy	AUC	F1	Precision	Recall
Random oversampling	0.844	0.904	0.759	0.695	0.837
SMOTE	0.846	0.896	0.749	0.719	0.782
Borderline SMOTE	0.836	0.898	0.734	0.702	0.769
SVM SMOTE	0.848	0.904	0.75	0.726	0.776
Modified SMOTE	0.834	0.905	0.758	0.663	0.884

As shown in Table 5, all the features and the modified SMOTE oversampling algorithm were used in this paper to compare common machine learning classifiers with the proposed SMSN classifier. Random forest had the highest precision, which was biased towards recognizing negative classes. However, its recall was the lowest, and its ability to identify positive classes was very poor. Logistic regression had the highest recall and the highest rate of recognizing positive classes, but its rate of recognizing negative classes was very low. The SMSN model had the highest Accuracy, AUC and F1. with Precision and Recall in the top three. Combined with the advantages of three base models, which were random forest, logistic regression, and Catboost, its performance was significantly improved.

**Table 5:** Classification experimental results of different machine learning classifiers

Classifiers	Accuracy	AUC	F1	Precision	Recall
Random forest	0.862	0.896	0.729	0.861	0.633
Logistic regression	0.834	0.905	0.758	0.663	0.884
Catboost	0.866	0.901	0.755	0.817	0.701
SVM	0.832	0.845	0.754	0.662	0.878
GBDT	0.862	0.892	0.745	0.815	0.687
XGBoost	0.858	0.888	0.732	0.822	0.66
LightGBM	0.872	0.893	0.763	0.837	0.701
SMSN	0.882	0.915	0.794	0.814	0.776

**Fig. 7:**
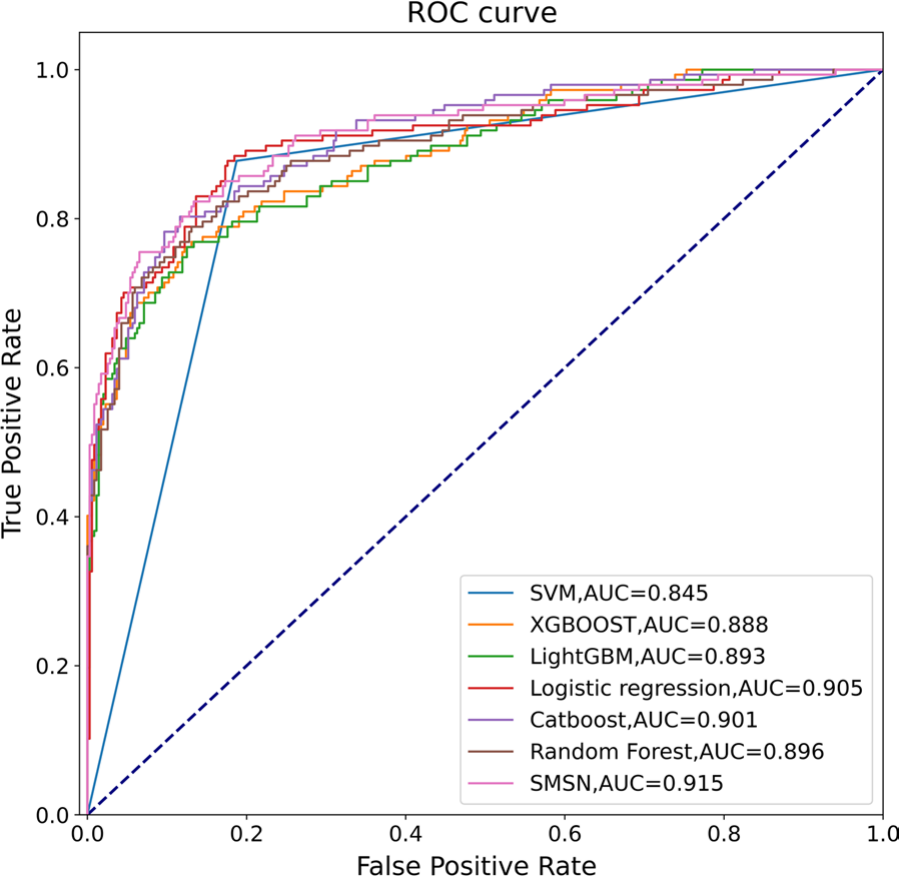
ROC curve of different classifiers

**Fig. 8:**
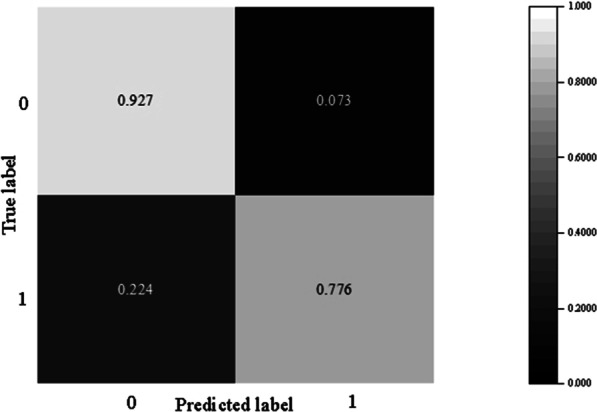
Confusion matrix of classifiers

The ROC curves of the different machine learning classifiers are shown in Fig. 7. The SMSN model had the largest ROC curve area, and its AUC was 0.915, which was larger than that of other machine learning classifiers. As shown in Fig. 8, the recall of the SMSN classifier was 0.776, which had an increase of 0.082 compared with the logistic regression classifier without the oversampling algorithm, indicating that the proposed oversampling algorithm could improve the rate of recognizing positive classes. In addition, the recall of the SMSN model was lower by 0.108 compared to that of the logistic regression classifier using the modified SMOTE algorithm. However, its precision was increased by 0.151, and all other indices performed better. The SMSN model achieved a balance in the recognition of positive and negative classes because its AUC and F1 were the highest.

Table 6 shows the prediction performance of traditional Apache scoring, which obtained mediocre reviews under complex clinical conditions. Compared with the Apache scoring, the SMSN classifier based on a machine learning algorithm has improved significantly because it considers more features and uses more data.

**Table 6:** Apache scoring experiment

Classifiers	Accuracy	AUC	F1	Precision	Recall
Apache	0.713	0.667	0.235	0.55	0.15
SMSN	**0.882**	**0.915**	**0.794**	**0.814**	**0.776**

The significance of bold mean that better performance results are obtained in this experiment

### Interpretability

**Fig. 9:**
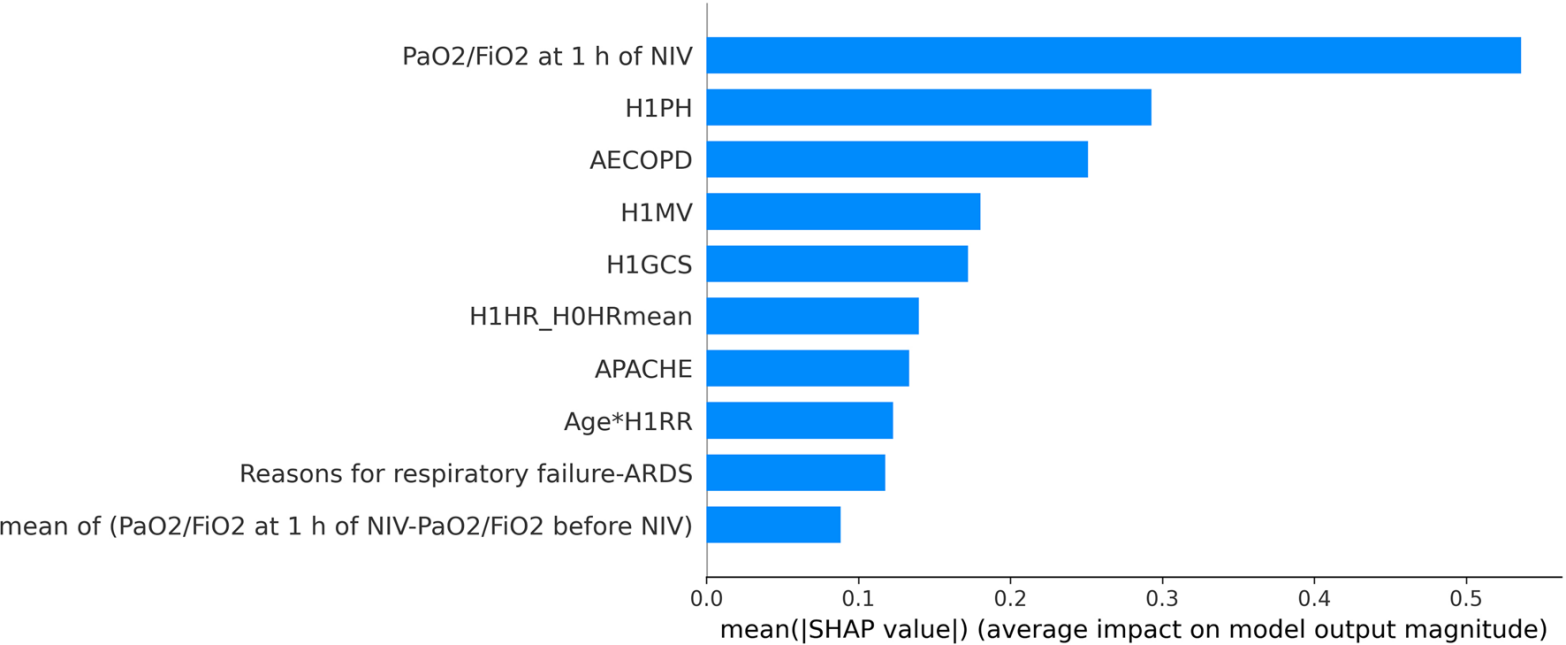
The feature importance of random forests based on SHAP

**Fig.10:**
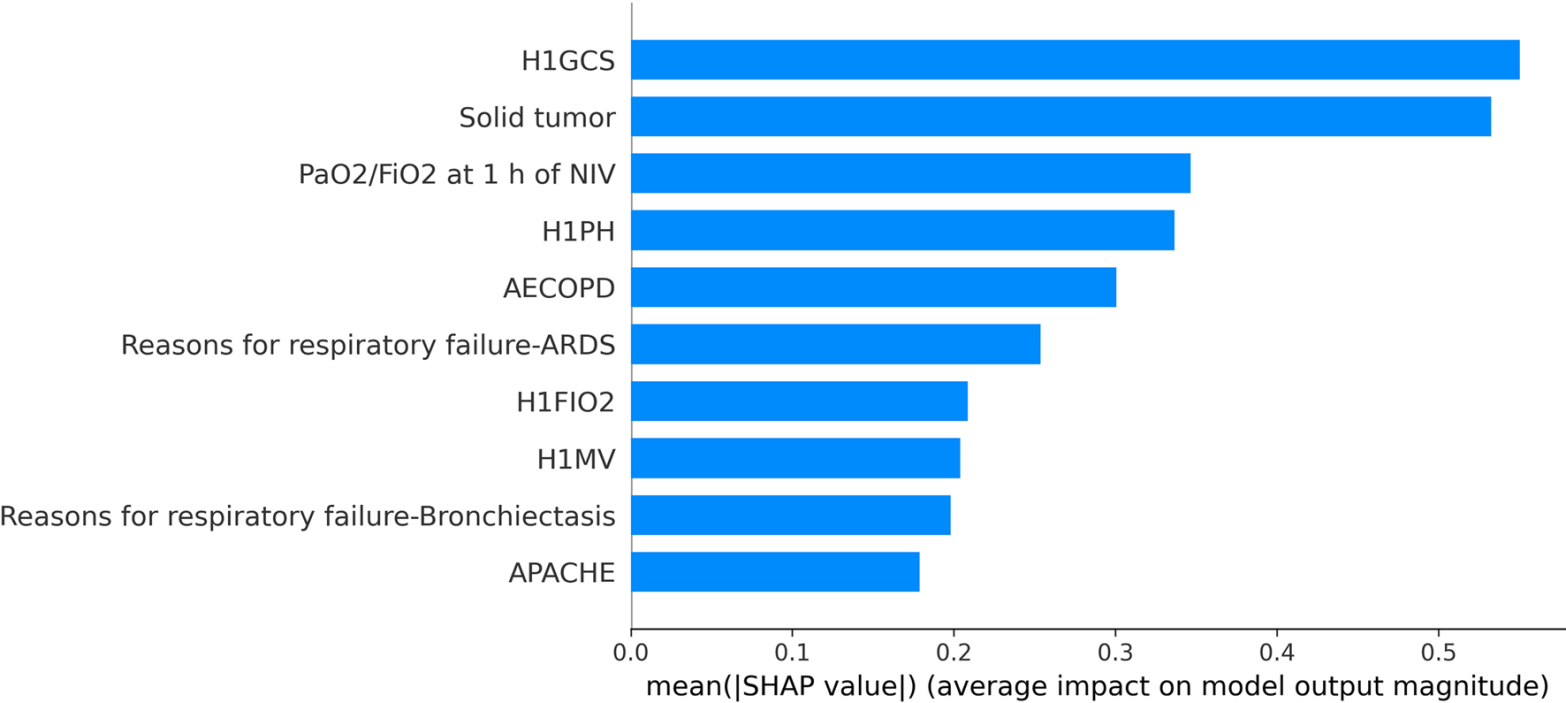
The feature importance of LightGBM based on SHAP

**Fig. 11:**
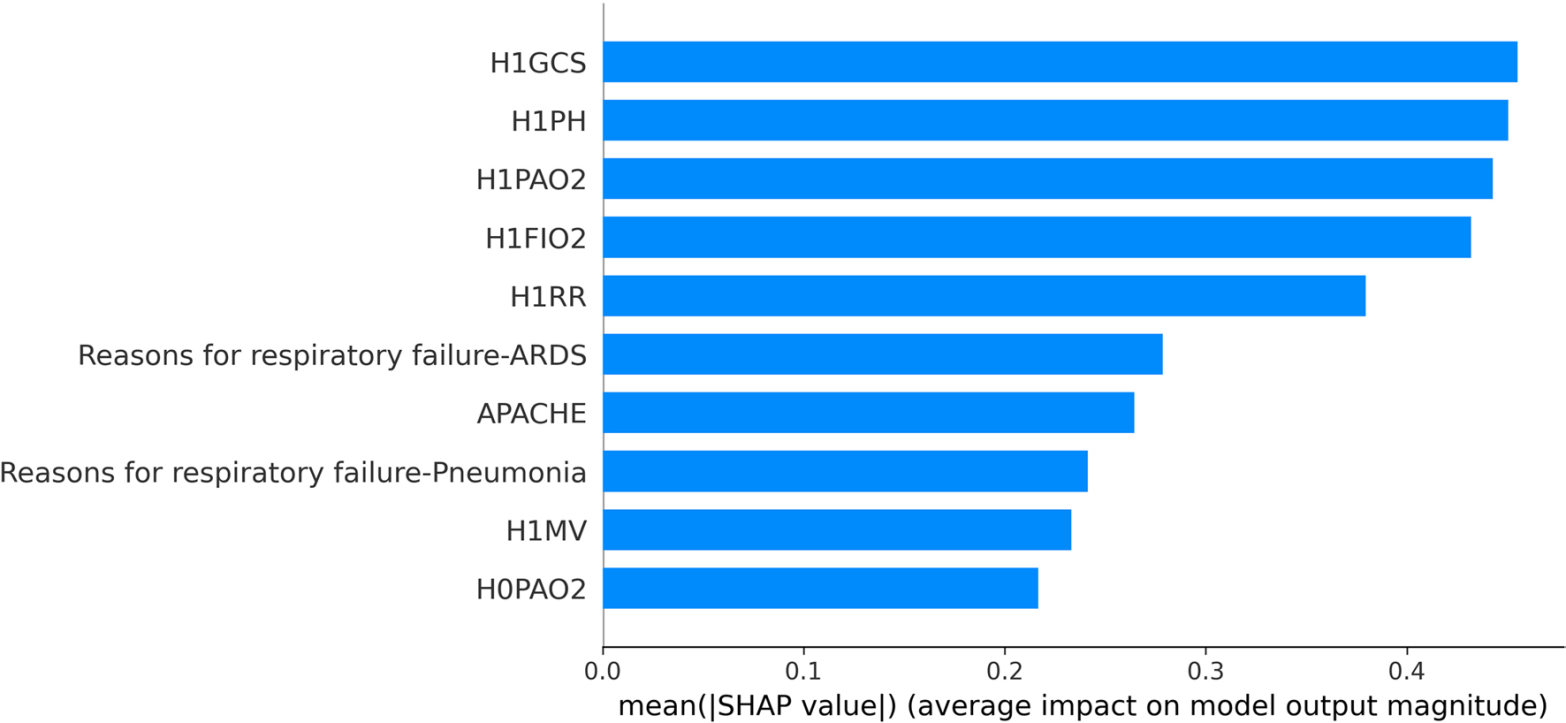
The feature importance of logistic regression based on SHAP

In this paper, SHAP was used to conduct feature importance analysis on the three base classifiers, and the results are shown in FigS. 9, 10 and 11. The top three features of SHAP values in the random forest classifier were PaO_2_/FiO_2_ at 1 h of NIV, H1PH and AECOPD. The top three features of SHAP values in the LightgBM classifier were H1GCS, solid tumour and PaO_2_/FiO_2_ at 1 h of NIV. The top three features of SHAP values in the logistic regression model were H1GCS, H1PH and H1PAO_2_. The top three features of SHAP values in different classifiers had differences and similarities.

In the random forest classifier, the feature importance of PaO_2_/FiO_2_ at 1 h of NIV was much higher than that of H1PH, and the first three features of SHAP values in the other two models had little difference. In the logistic regression model, the SHAP values of PaO_2_/FiO_2_ at 1 h of NIV even couldn’t enter the top ten, which was very different from the random forest model. Three classifiers were used for prediction, and the features valued by the classifiers were significantly different and had their own characteristics. The stacking ensemble algorithm could combine the advantages of different classifiers to obtain better predictions.

**Fig. 12:**
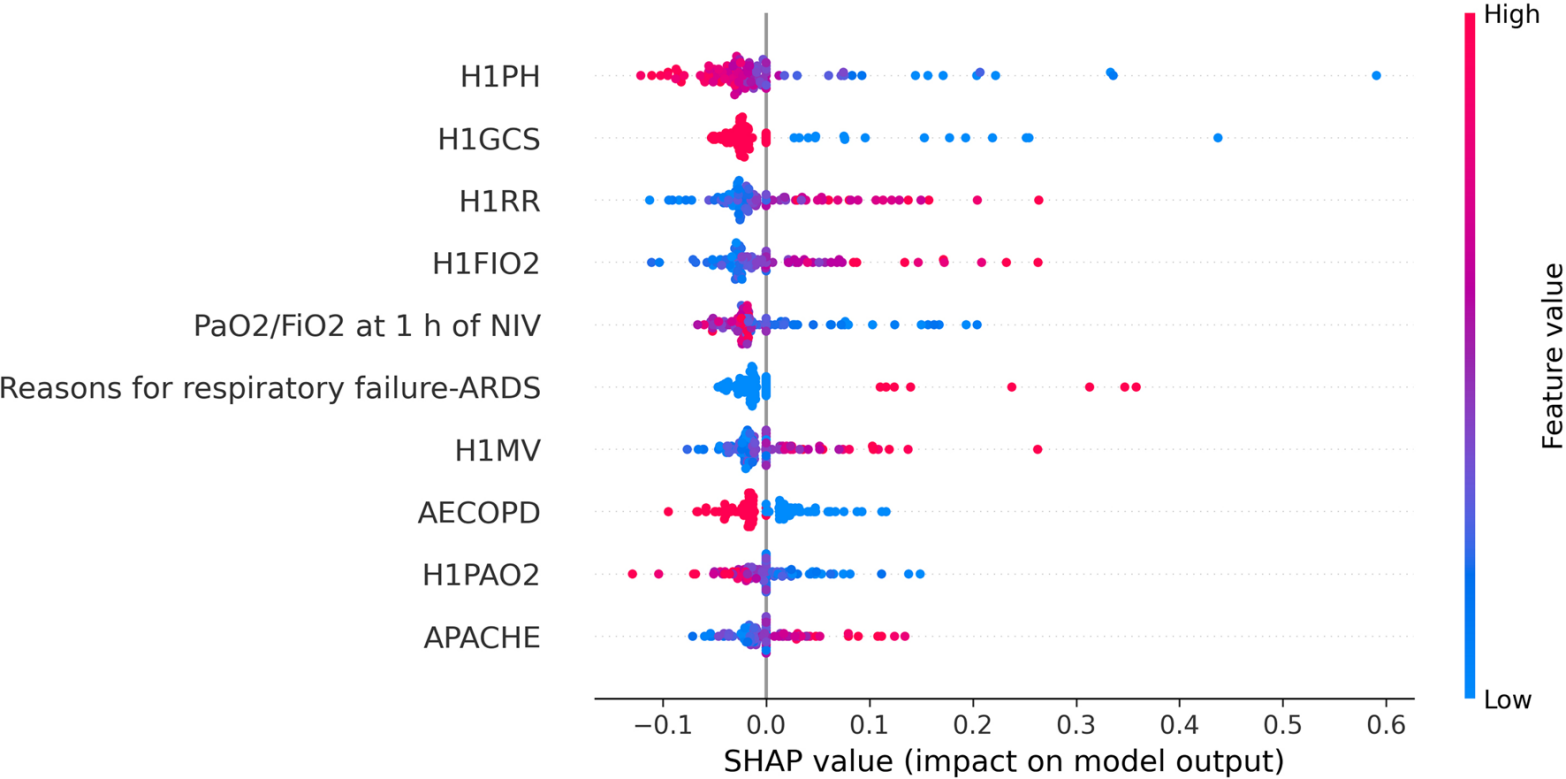
Analysis of SHAP values of the SMSN model

Figure 12 is the visual figure of the SHAP values of the SMSN, which was sorted according to the average SHAP values, and the higher the feature was, the larger the average SHAP value was. The horizontal axis in the figure represents the SHAP value, and each feature has many points on the horizontal axis to represent the sizes of the SHAP values of the instance in the testing dataset. In addition, the colours of these points represent the feature values. Dark blue indicates that the feature value was small, and dark red indicates that the feature value was large.

H1PH, H1GCS, H1FIO_2_, H1RR and PaO_2_/FiO_2_ at 1 h of NIV of the average values of features were found to have the highest SHAP values. Among them, the higher the feature values of H1PH, H1GCS and PaO_2_/FiO_2_ at 1 h of NIV were, the smaller their SHAP values were, and the lower the probability of patients’ noninvasive ventilation failure was. Furthermore, the higher the feature values of H1FIO_2_, H1RR and reasons for respiratory failure-ARDS were, the larger their SHAP values were, and the higher the probability of patients’ noninvasive ventilation failure was.

**Fig. 13:**
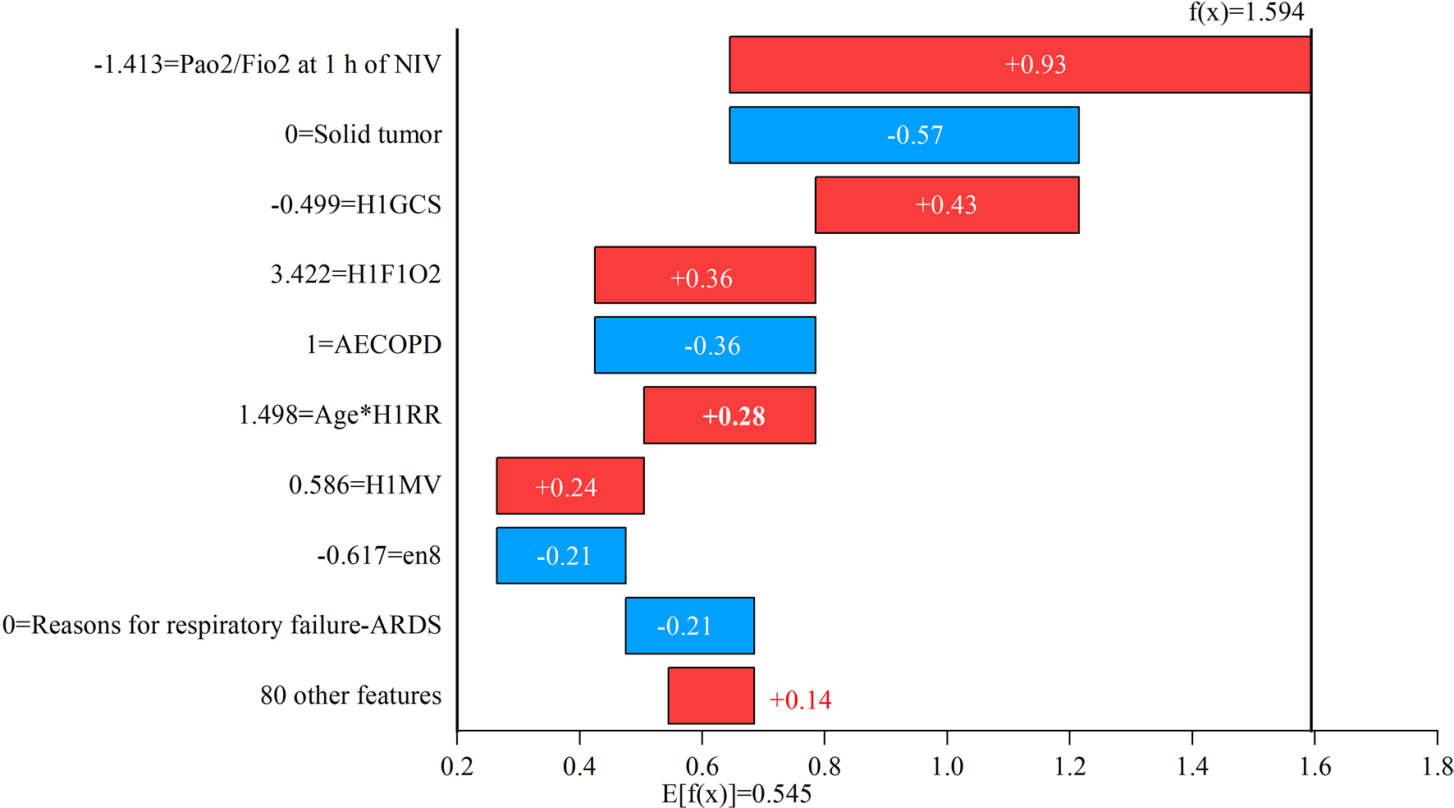
SHAP analysis of the noninvasive ventilation failure patient I

**Fig. 14:**
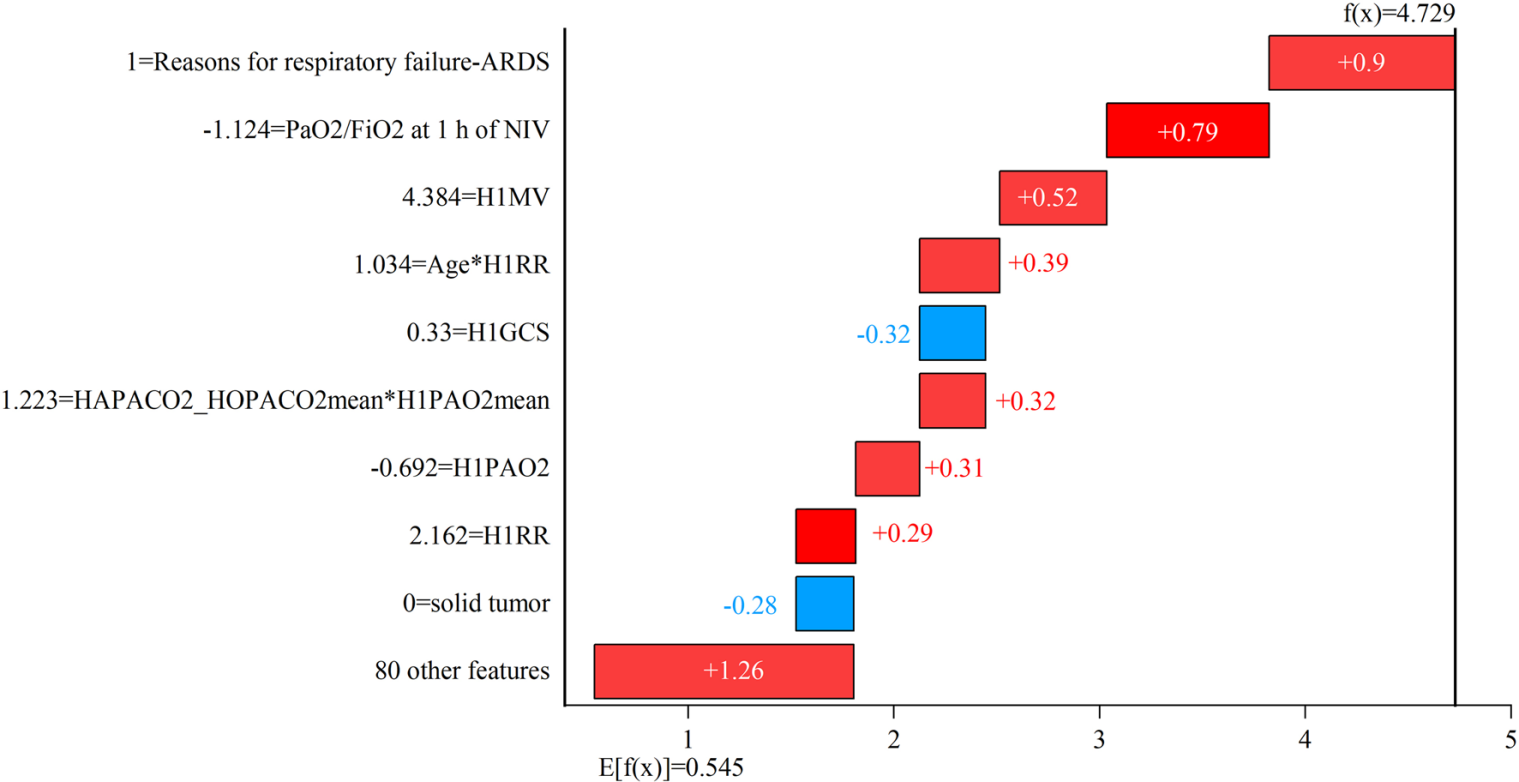
SHAP analysis of the noninvasive ventilation failure patient II

As shown in Figs. 13 and 14, taking the random forest classifier as an example, SHAP analysis was used for the predictions of two patients who failed noninvasive respiratory therapy. E[*f*(*X*)] = 0.545 represents the average predicted values of the instance in the test set. In Fig. 13, f(*X*) = 1.594 represents the predicted value of patient I who failed noninvasive respiratory therapy, which was higher than the average predicted value of 1.049. According to the SHAP formula, the sum of the SHAP values of 89 features was 1.049. As shown in Fig. 13, PaO_2_/FiO_2_ at 1 h of NIV was low, leading to a high SHAP value. However, the patient’s solid tumour was 0, resulting in a negative SHAP value. The age*H1RR feature was generated according to expert knowledge, and its SHAP value was positive. As shown in Fig. 14, the reason for respiratory failure—ARDS—was 1, and PaO_2_/FiO_2_ at 1 h of NIV was low, which made the SHAP value high. Compared with Fig. 13, the H1GCS value was higher in Fig. 14, and its SHAP value was negative, while the SHAP was positive in Fig. 13. Even if the predicted results all failed, the SHAP values of the same feature were positive and negative, with a large difference. Moreover, the decision-making process of two patients to obtain the same prediction result was different, and SHAP could be used as a tool for clinicians to interpret the prediction process.

**Fig. 15:**
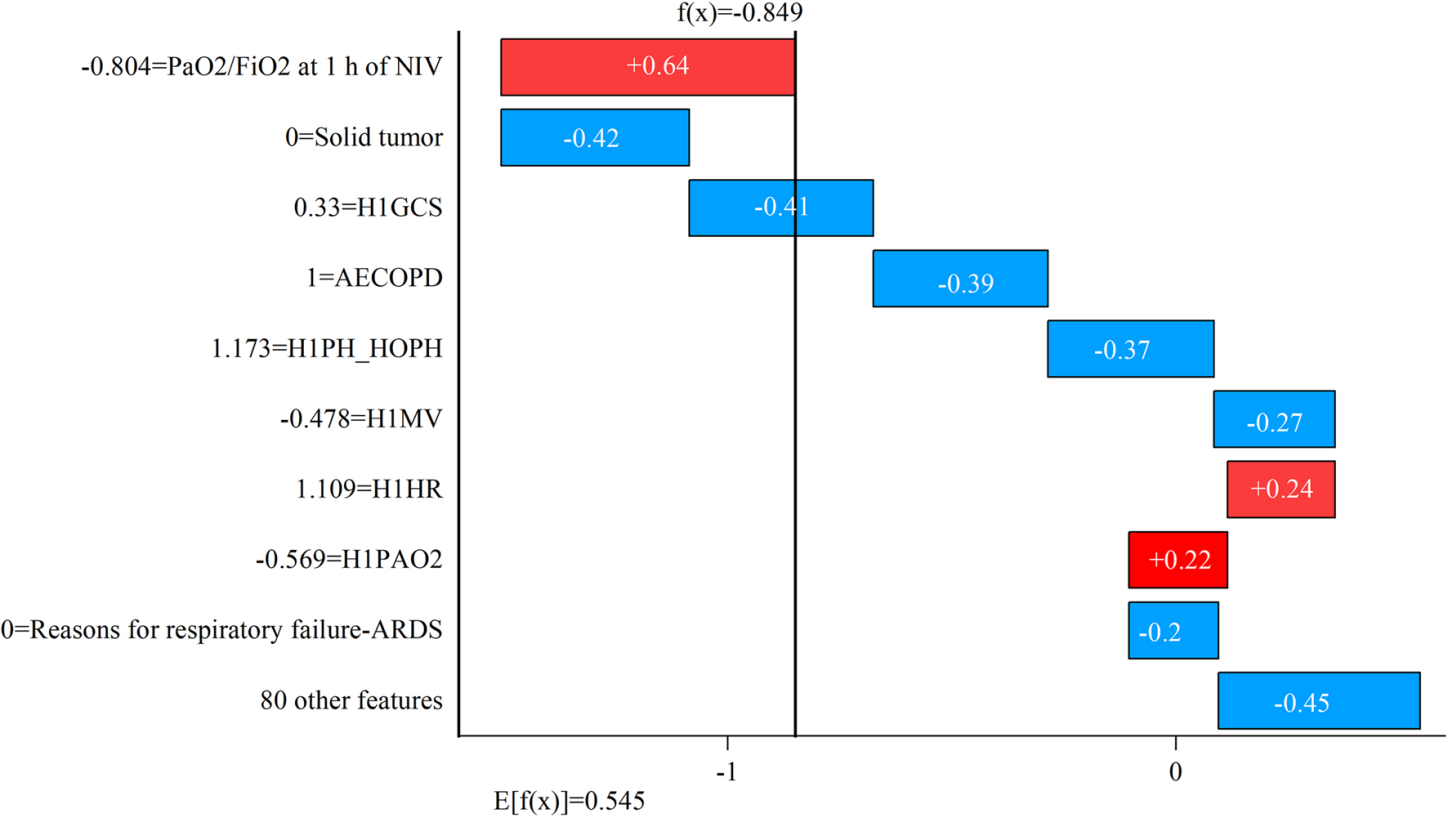
SHAP analysis of successful patient I with noninvasive respiratory therapy

As shown in Fig. 15, although the patient had a low PaO_2_/FiO_2_ at 1 h of NIV, he had no solid tumour, no ARDS, and a high H1GCS, which made the failure probability of the patient predicted by the final model low. It can be observed that SHAP’s interpretation of the SNSM’s predictions helped doctors make decisions.

## Discussion

There were data related to time series in the collected noninvasive respiratory data. When the LSTM-based autoencoder model was used to extract time series features, both accuracy and F1 were improved. Traditional machine learning algorithms are unable to handle data imbalance well, resulting in a very low recall. When the proposed modified SMOTE algorithm was introduced, the recognition ability of positive classes was enhanced and recall was significantly improved, but its AUC and F1 were still not high. To further improve the performance of the classifier, the idea of ensemble learning was introduced, and the stacking ensemble algorithm was adopted. In addition, three different machine learning classifiers were combined to obtain the SMSN model. Through experimental verification, the SMSN classifier was optimal in AUC, F1 and accuracy performance measures. Additionally, taking the components in the SMSN model, namely, the logistic regression classifier, as an example, SHAP was used to analyse the decision path of each patient’s prediction result, i.e., how each feature affected the final prediction. Through the proposed SMSN model, the failure probability of noninvasive ventilation could be predicted more accurately before the use of noninvasive ventilation or in the early stage (1–2 h) of noninvasive ventilation. Accordingly, recommendations were made for patients with a high risk of failure to help doctors decide when to escalate or change the treatment plan, which was of scientific significance for the decision support of doctors with low seniority to make decisions.

## Data Availability

The use of data in this study is limited, and the data set can be obtained from the corresponding author (Jun Duan) according to reasonable requirements.

## Abbreviations

SMSN: Stacking and modified SMOTE algorithm of prediction of noninvasive
LSTM: Long short-term memory
ICU: Intensive care unit
SMOTE: Synthetic minority oversampling technique
NIV: Noninvasive ventilation
COPD: Chronic obstructive pulmonary disease
EMR: Electronic medical record
DUNs: Deep unified networks
GCS: Glasgow coma score
AUC: Area under curve
ROC: Receiver operator characteristic curve
KNN: K-nearest neighbor
SVM: Support vector machines
K-Means: Clustering algorithm
